# Renal glucose release during hypoglycemia is partly controlled by sympathetic nerves – a study in pigs with unilateral surgically denervated kidneys

**DOI:** 10.14814/phy2.12603

**Published:** 2015-11-12

**Authors:** Sabine J Bischoff, Martin Schmidt, Thomas Lehmann, Matthias Schwab, Georg Matziolis, Alexander Saemann, René Schiffner

**Affiliations:** 1 Institute for Laboratory Animals and Welfare, Jena University HospitalJena, Germany; 2 Institute for Biochemistry II, Jena University HospitalJena, Germany; 3 Institute of Medical Statistics, Computer Sciences and Documentation Science, Jena University HospitalJena, Germany; 4 Department of Neurology, Jena University HospitalJena, Germany; 5 Orthopaedic Department, Jena University HospitalJena, Germany; 6 Department of Internal Medicine II, Helios HospitalErfurt, Germany

**Keywords:** Glucose release, hypoglycemia, renal denervation, side dependent

## Abstract

Catecholamines are known to increase renal glucose release during hypoglycemia. The specific extent of the contribution of different sources of catecholamines, endocrine delivery via circulation or release from autonomous sympathetic renal nerves, though, is unknown. We tested the hypothesis that sympathetic renal innervation plays a major role in the regulation of renal gluconeogenesis. For this purpose, instrumented adolescent pigs had one kidney surgically denervated while the other kidney served as a control. A hypoglycemic clamp with arterial blood glucose below 2 mmol/L was maintained for 75 min. Arteriovenous blood glucose difference, inulin clearance, *p*-aminohippurate clearance, and sodium excretion were measured in intervals of 15 min separately for both kidneys. Blood glucose was lowered to 0.84 ± 0.33 mmol/L for 75 min. The side-dependent renal net glucose release (SGN) decreased significantly after the unilateral ablation of renal nerves. In the linear mixed model, renal denervation had a significant inhibitory effect on renal net glucose release (*P* = 0.036). The SGN of the ablated kidney decreased by 0.02 mmol/min and was equivalent to 43.3 ± 23.2% of the control (nonablated) kidney in the pigs. This allows the conclusion that renal glucose release is partly controlled by sympathetic nerves. This may be relevant in humans as well, and could explain the increased risk of severe hypoglycemia of patients with diabetes mellitus and autonomous neuropathy. The effects of denervation on renal glucose metabolism should be critically taken into account when considering renal denervation as a therapy in diabetic patients.

## Introduction

The ablation of autonomous nerves, which are attached to the adventitia of renal arteries, is a new interventional method to treat hypertension (Schmieder et al. 2012). The pathophysiological correlate of this intervention might be suppression of renal renin release, which is the key stimulus for the renin–angiotensin–aldosterone hormone system. The classical indication of renal denervation comprises resistant hypertension, which is frequently associated with metabolic alterations such as overweight and obesity, impaired fasting glucose, and impaired glucose tolerance, as well as insulin resistance. Sympathetic activation has been clearly identified as an important contributing factor to this detrimental clinical scenario (Lambert et al. 2010; Smith and Minson 2012). Furthermore, the sympathetic nervous system has a pathophysiological involvement in many other diseases as well, for example, arterial hypertension, sleep apnea syndrome (Witkowski et al. 2011), chronic heart failure (Davies et al. 2013), chronic kidney disease (Hering et al. 2012b), and polycystic ovary syndrome (Sverrisdottir et al. 2008). Hence, new indications for this promising technique of renal denervation may arise in the near future. In regard to the literature, however, only a few smaller studies have been published and no long-term results are existent (Davis et al. 2013). On the other hand, the use of this new technology and the results of renal denervation are being viewed controversially. In a blinded study no significant reduction in systolic blood pressure compared to a sham procedure was observable after 6 months (Bhatt et al. 2014); however, it was not proven that an actual renal denervation had been achieved in the patients of the study. The expert opinion in regard to the use of renal denervation as a therapeutic treatment is increasingly guarded, and in addition, the treatment of other diseases with this procedure is viewed as being experimental (Schlaich et al. 2013). From a pathophysiological point of view, activation of the sympathetic nervous system may be related to the metabolic syndrome and the glucose metabolism (Noll et al. 1996; Sverrisdottir et al. 2008; Lambert et al. 2010; Parati and Esler 2012; Kiuchi et al. 2013).

Therefore the question arises, what kind of side effects a method that changes sympathetic innervation has on the glucose metabolism in the kidneys? There is good evidence that endogenous catecholamines from the circulation and other hormones stimulate renal glucose release (Cersosimo et al. 1997) – the effect of the autonomous nervous system on renal gluconeogenesis, however, is, hitherto, only incompletely understood (Triplitt 2012). Our hypothesis is that the glucose metabolism is altered by renal denervation.

Recent studies have documented the reduction of peripheral insulin resistance in type 2 diabetic patients as a side effect of renal denervation (Mahfoud et al. 2011). A possible pathophysiological mechanism for this observation might be that renal gluconeogenesis is not only stimulated hormonally by catecholamines, but also by autonomous sympathetic nerves. Renal nerve ablation might inhibit renal glucose release and therefore reduce insulin resistance (Straznicky et al. 2011; Hering et al. 2012a). This, however, would have multiple implications, including the increased risk of severe hypoglycemia in patients with antidiabetic medication. Endogenous gluconeogenesis is important for the human blood glucose homeostasis; while the liver contributes about 70%, the kidneys add 30% to human gluconeogenesis (Meyer et al. 1999). As a result, the risk of severe hypoglycemia increases tremendously in patients with antidiabetic medication and renal impairment. This fact limits near-normoglycemic metabolic control in type 1 and type 2 diabetes (Morales and Schneider 2014).

We tested the hypothesis that the ablation of renal autonomous nerves decreases renal gluconeogenesis in pigs because the blood glucose homeostasis of humans and pigs has many common aspects (Miller and Ullrey 1987). The ablation of one kidney allows the other kidney to be used as a control in the same animal. Severe hypoglycemia is a strong stimulus to enhance endogenous gluconeogenesis and can be performed in deep general anesthesia.

## Methods

### Experimental animals and surgical procedures

Surgery was performed on nine female pigs, weighing between 35 and 45 kg, and belonging to the breed “German land race” pig, in strict accordance to local standards and the “Guide for the Care and Use of Laboratory Animals” (Care & and Use of Laboratory Animals, 2011). The local ethics committee’s approval was obtained for this study. After food withdrawal for 24 h, surgery was performed in the supine position under sterile conditions and under general anesthesia. First, the pigs were anesthetized by administering an intramuscular injection of 15 mg/kg ketamine hydrochloride (Ketavet®, 100 mg/mL, Pharmacia Upjohn, Erlangen, Germany) and 0.2 mg/kg midazolam hydrochloride (Midazolam-ratiopharm®, Ulm, Germany). Thereafter, 0.2–0.3 mg/kg propofol (Disoprivan®, AstraZeneca, Wedel, Germany) was administered through a venous catheter in the ear vein (Vasocan®, Braun Melsungen, Melsungen, Germany) and the pigs were intubated orotracheally (Trachealtubus, Rüsch, Kernen, Germany). Anesthesia was maintained by continuous inhalation of 1.5% isoflurane (Isofluran®, DeltaSelect, Dreieich, Germany) and O_2_. Analgesia was achieved through the intravenous application of 0.003 mg/kg fentanyl per hour (0.05 mg/mL Fentanyl, Janssen). Muscular relaxation was induced by administration of up to 0.1 mg/kg pancuronium (Pancuronium-Actavis®, Actavis, München, Germany). The cornea was kept moistened during anesthesia by administering eye drops (Corneregel®, Bausch&Lomb, Berlin, Germany). During anesthesia, a controlled stable blood pressure was maintained by intravenous administration of isotonic saline (Isotonische Kochsalzlösung®, Fresenius, Bad Homburg, Germany). Vascular catheters (Arteriofix®, Braun, Melsungen, Germany) were inserted into the carotid artery for blood sampling and blood pressure measurement, and into the jugular vein for intraoperative administration of drugs and fluid infusion. After a midline laparotomy and opening of the retroperitoneum, catheters with six Charier were inserted (Actreen® Glys Cath, Braun, Melsungen, Germany) in both ureters for urine sampling. Both renal veins were instrumented using vascular catheters (Certofix® Trio, Braun, Melsungen, Germany) for local blood sampling, which were fixed with Liquiband® Flow Control (Advanced Medical Solution, Devon, UK). Thereafter, the complete plexus renalis was removed unilaterally (randomized, see below). Euthanasia was performed after the termination of the experiments via intravenous administration of 60 mg/kg pentobarbital (Narcoren®, Merial, Hallbergmoos, Germany).

The side of the unilateral denervation of the renal arteries was determined by using a randomization list. Denervation was performed by surgical removal of the adventitia of the renal arteries. An open abdomino-retroperitoneal entrance was used for surgery.

### Induced polyuria and maintenance of blood pressure

A polyuria of more than 100 mL/h per kidney was of crucial importance to the experiment. Exogenous catecholamines for blood pressure maintenance were not administered in order to avoid any bias. Instead, high-volume infusions of sterile isotonic saline were administered via a central venous catheter in order to induce a hyperperfusion of the kidneys. The infusion rate varied between 1 and 2 L/h and was adjusted according to the invasively measured blood pressure and the urine production. Additionally, furosemide (Furosemid-ratiopharm®, 40 mg/mL, Ratiopharm, Ulm, Germany) was administered if the urine volume decreased. Necessary dosage of furosemide varied within the range of 40–120 mg per animal.

### Hypoglycemic clamp

An insulin-induced hypoglycemia was maintained below 2 mmol/L (down to 0.5 mmol/L) over 75 min, starting with a bolus of 15 IU human regular insulin (Actrapid®, Penfill®, 100 IU/mL, Novo Nordisk Pharma, Mainz, Germany) administered via a central venous catheter. A commercially available blood glucose meter (Contour®, Bayer AG, Leverkusen, Germany) was used to monitor the arterial blood glucose level every 7.5 min. Additional boluses of regular insulin were administered if necessary. The individual total insulin dosages varied between 20 and 100 IU of insulin per experiment.

### Determination of side-dependent glomerular filtration rate, renal plasma flow, and gluconeogenesis

Concentrations of *p*-aminohippurate (PAH) (Sigma–Aldrich, Taufkirchen, Germany) and inulin (Sigma–Aldrich, Taufkirchen, Germany) in the blood plasma were brought to near equilibrium using a two-step protocol. As an initial bolus, 3.1 g PAH and 0.55 g inulin were dissolved in 500 mL sterile isotonic saline at a temperature of 70–80°C. This solution was cooled down to a temperature of 30–37°C and was administered via a central venous catheter over a period of 30 min. The maintenance solution was prepared using 4.3 g PAH and 1.1 g inulin dissolved in 1000 mL sterile isotonic saline. This maintenance infusion was continuously infused at a rate of 250 mL/h until the end of the experiment.

The side-dependent renal plasma flow (SRP) was determined separately for each kidney, using the equation: SRP [mL/min] = *C*_UrinePAH_ [mg/L] × *V*_UrineVolumeOverTime_ [mL/min]/*C*_PlasmaPAH_ [mg/L]/0.9 (Reubi 1953; Constanzo 2007). Urine specimens for the analysis of *C*_UrinePAH_ were collected via catheters from both ureters. For the analysis of *C*_PlasmaPAH_, blood specimens were drawn from the carotid artery. Urine and blood specimens were collected every 15 min. *V*_UrineVolumeOverTime_ was determined using the ureter catheters. The urine of each kidney was collected in intervals of 15 min, with the volumes measured respectively. The glomerular filtration rate (GFR) was determined separately for each kidney, using the equation: *C*_UrineInulin_ [mg/L] × *V*_UrineVolumeOverTime_ [mL/min]/*C*_PlasmaInulin_ [mg/L] (Soveri et al. 2014). Urine specimens for the analysis of *C*_UrineInulin_ were collected via catheters from both ureters. For the analysis of *C*_PlasmaInulin_, blood specimens were drawn from the carotid artery. Urine of each kidney and blood specimens were collected every 15 min. *V*_UrineVolumeOverTime_ was determined using the ureter catheters. The side-dependent renal glucose release (SGN) was calculated separately for each kidney, using the equation: SGN [mmol/min] = SRP [L/min] × (*C*_VenousGlucose_ [mmol/L] − *C*_ArterialGlucose_ [mmol/L]). *C*_VenousGlucose_ was measured from blood specimens from catheters in both renal veins. Blood specimens for the analysis of *C*_ArterialGlucose_ were collected from a catheter in the carotid artery.

### Preanalytical methods

Plasma and urine samples aliquots were collected and stored at −20°C up to their analysis. For the measurement of *p*-aminohippuric acid (PAH), samples were thawed in a water bath at 37°C, vortexed, and centrifuged at 18,000 ***g*** for 5 min at room temperature in a microcentrifuge. The cleared supernatants were used for further analyses. Reagents were of analytical grade (purchased from Roth, Karlsruhe, Germany) and were dissolved and diluted in MilliQ-water, if not indicated otherwise.

### Quantitation of inulin

The method described by Roe et al. was adapted to a format suitable for measurements in microplate readers (Roe et al. 1949). Supernatants (250 *μ*L) from the preclearing step were deproteinized by sequential addition of 250 *μ*L ZnSO_4_ × 7H_2_O (10% solution), 250 *μ*L NaOH (0.5 N), and 500 *μ*L water, with intense vortexing after each addition. The samples were then again centrifuged for 10 min. For the initial analysis, the deproteinized supernatants were used undiluted (plasma) or routinely diluted 1:10 (urine). A portion of the samples needed additional dilution to stay within the standard range, depending on the animals and the sites of sample taking. Inulin standard curves were generated from a stock solution of 7.5 mg inulin per mL, which was solubilized by microwaving at 200 W for 5 sec. About 250 *μ*L of standards (50, 25, 12.5, 6.25, 1.6, and 0 *μ*g inulin per mL) or deproteinized supernatants were mixed with 125 *μ*L resorcine reagent (0.1 g resorcine and 0.25 g thiourea dissolved in 100 mL glacial acetic acid). After the addition of 1 mL of HCl (30%), all samples were kept in the dark for the subsequent steps: incubation for 20 min at 80°C in a controlled heating block, cooling in an ice/water bath, transfer of three 200 *μ*L replicates from each sample to a microwell plate, shaking for 10 sec, and absorbance measurement at 546 nm in a microplate reader. For six consecutive assays, this protocol allowed for a lower limit of detection of 1.8 ± 1.0 *μ*g inulin per mL of undiluted sample (means ± SD; *n* = 6, applies also to numerical values given below). Linear calibration lines were highly reproducible with correlation coefficients of 0.998 ± 0.001. The precision was 4.6 and 6.5% (coefficients of variation) and the accuracy (recovery) was 96 and 98% for solutions containing 12.5 *μ*g or 25 *μ*g inulin per mL.

### Quantitation of PAH

Essentially, the method described by Agarwal et al. was applied (Agarwal 2002). This microplate assay based on the reaction of *p*-dimethylaminocinnamaldehyde (Sigma–Aldrich, Taufkirchen, Germany) with PAH gives results that are in very good agreement with HPLC-based methods. For plasma, the precision was 2.5 and 3.2%, and the accuracy was 98 and 100% for solutions containing 12 *μ*g or 24 *μ*g PAH per mL. For urine, the precision was 2.8 and 4.1% and the accuracy was 100 and 97% for solutions containing 30 *μ*g or 60 *μ*g PAH per mL.

### Quantitation of sodium excretion

The sodium concentration in urine samples was measured using a routine clinical procedure.

### Statistical analyses

Descriptive statistics (means ± standard deviation [SD]) were used to summarize the outcome parameters of the different measurements. A linear mixed effects model was adapted to compare the renal gluconeogenesis of the ablated and the nonablated side after hypoglycemia. In this analysis, side and measurement number are modeled as fixed effects and heterogeneity of the animals is incorporated by random effect. To detect possible differences in the progression of both sides, an interaction term of side and measurement number has also been included in the model. The significance level was set to *α *= 0.05. All analyses were performed with IBM SPSS Statistics19.0 (Armonk, NY).

## Results

**Figure 1:**
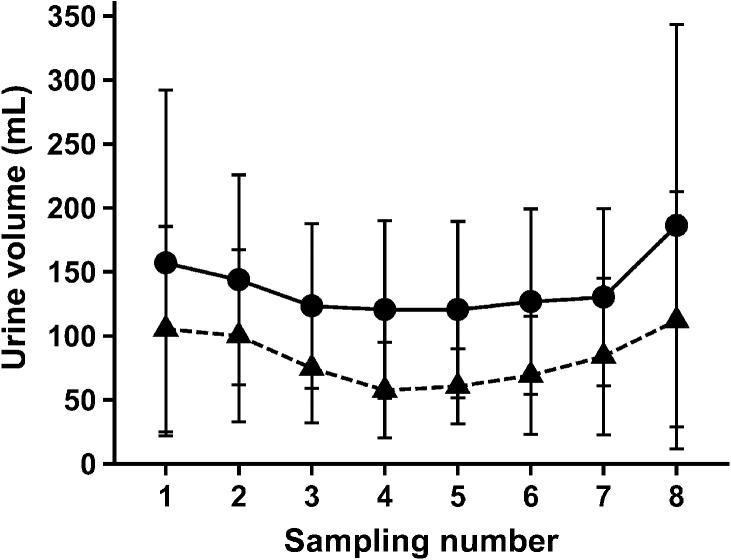
Side-dependent urine volume over time after unilateral ablation of renal nerves. No differences in the side-dependent urine volume (mL) during hypoglycemic clamp after the unilateral ablation of the renal nerves in the nonablated (●, line) and ablated (▲, dashed line) kidneys. Side-dependent urine production was measured during a high-volume infusion inducing hyperperfusion of the kidneys. Samples for measurements were taken every 15 min; means ± SD, *n* = 9, *P* > 0.05.

**Figure 2:**
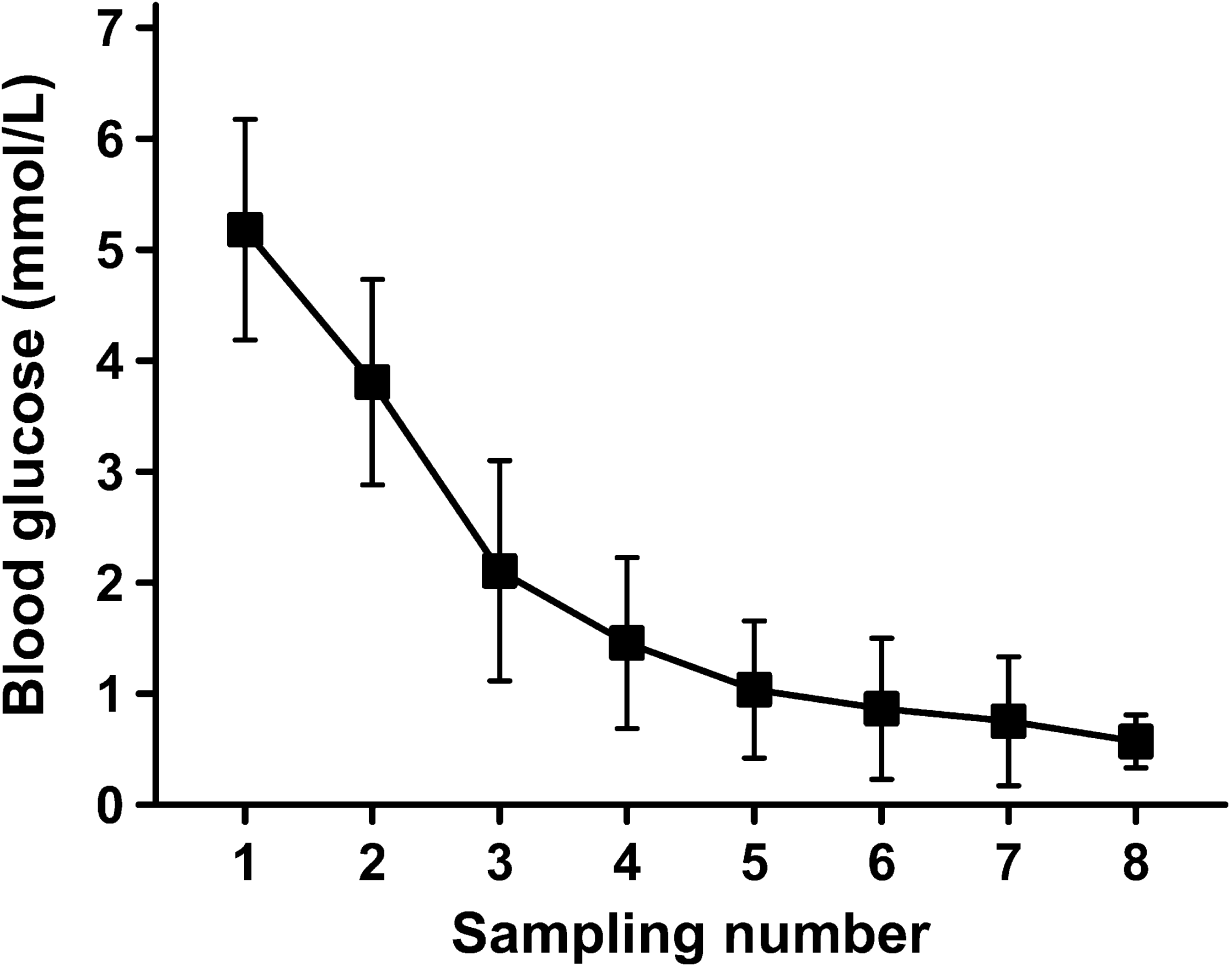
Mean blood glucose during hypoglycemic clamp after unilateral ablation of renal nerves. Mean blood glucose (mmol/L) measurements were performed every 15 min, starting with the application of insulin; means ± SD, *n* = 9.

To test our hypothesis that the glucose metabolism is altered by renal denervation we used pigs as an experimental model. After the renal denervation, a hypoglycemia was induced and maintained via dosages of insulin. In this experimental setting there was no difference in the side-dependent urine volume after the unilateral ablation of the renal nerves over time (Fig.1). Heart rate, blood pressure, and body temperature were maintained within normal range during the experiments (Table1). The mean blood glucose decreased from 5.9 ± 0.9 mmol/L to 0.84 ± 0.33 mmol/L. Hypoglycemia was considered to be established at blood glucose concentrations below 2 mmol/L and was maintained for 75 min (Fig.2). As the effective time of the establishment of hypoglycemia differed slightly between animals, all subsequent measurements in each individual animal refer to the first sampling in which the blood glucose was below the threshold of 2 mmol/L.

**Table 1:** Comprehensive data on the status of the animals during the experimental procedure

Variables	Parameters
Sex (*n*_male_/*n*_female_)	0/9
Weight (kg)	38 ± 7
Age (days)	85 ± 10
Heart rate at baseline (bpm)	111 ± 7
Heart rate during hypoglycemia (bpm)	124 ± 15
Blood pressure at baseline (systolic_mmHg_/diastolic_mmHg_)	109/75 ± 9/8
Blood pressure during hypoglycemia (mmHg) (systolic_mmHg_/diastolic_mmHg_)	102/68 ± 10/7
Body temperature at baseline (°C)	37.5 ± 0.5
Body temperature during hypoglycemia (°C)	36.1 ± 0.7

Data are given as means ± SD, *n* = 9.

**Figure 3:**
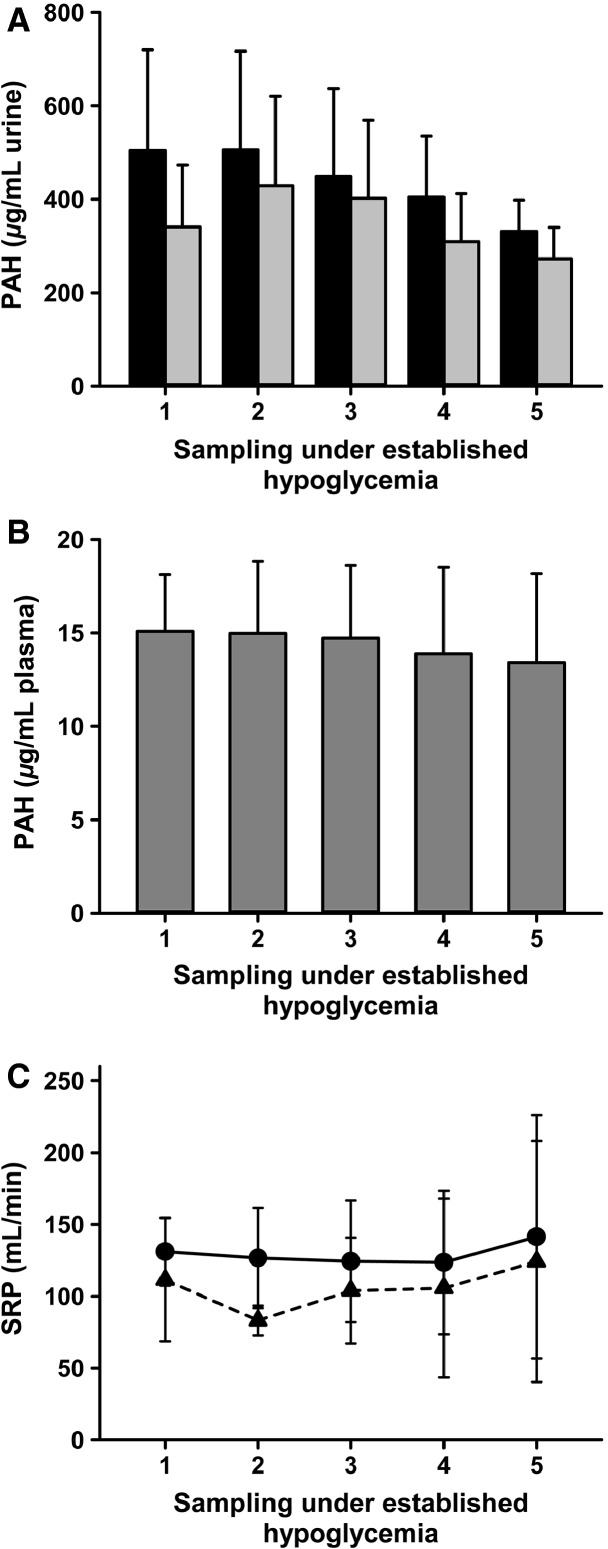
Side-dependent renal plasma flow (SRP) and *p*-aminohippurate (PAH) after unilateral ablation of the renal nerves. Samples for PAH measurements were taken every 15 min during high-volume infusion induced hyperperfusion of the kidneys after hypoglycemia (blood glucose below 2 mmol/L) was established. (A) No differences in PAH (*μ*g/mL] in urine during severe hypoglycemia after unilateral ablation of renal nerves between renal nerve ablated kidneys (black bars) and nonablated kidneys (gray bars); means ± SD, *n* = 9, *P* > 0.05. (B) Plasma PAH concentrations in samples drawn from the carotid artery during severe hypoglycemia are well below the saturation concentration for the tubular secretion system; means ± SD, *n* = 9. (C) No differences in SRP (mL/min) during severe hypoglycemia after unilateral ablation of renal nerves in nonablated (●, line) and ablated (▲, dashed line) kidneys; means ± SD, *n* = 9, *P* > 0.05.

**Figure 4:**
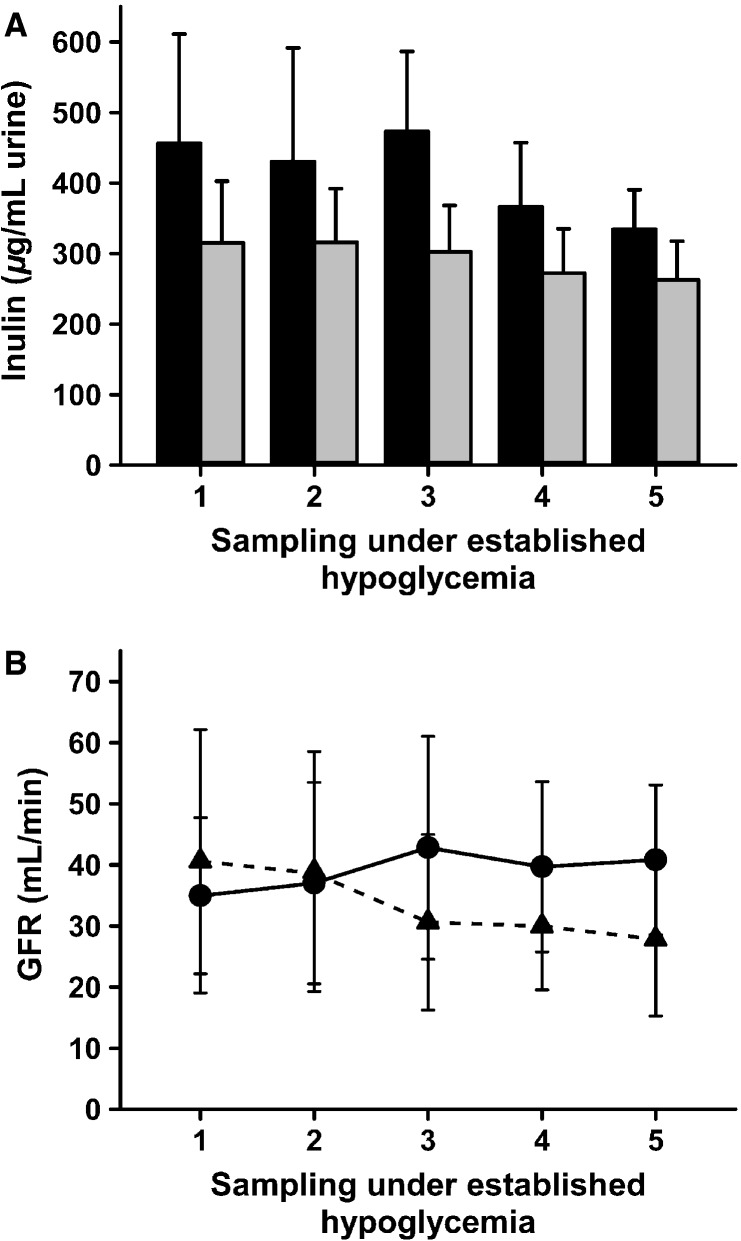
Glomerular filtration rate (GFR) and inulin after unilateral ablation of renal nerves. Samples for GFR measurements were taken every 15 min during high-volume infusion induced hyperperfusion of the kidneys after hypoglycemia (blood glucose below 2 mmol/L) was established. (A) No differences in inulin (*μ*g/mL) during severe hypoglycemia after unilateral ablation of renal nerves between renal nerve ablated kidneys (black bars) and nonablated kidneys (gray bars); means ± SD, *n* = 9, *P* > 0.05. (B) No differences in GFR (mL/min) during severe hypoglycemia after unilateral ablation of renal nerves in nonablated (●, line) and ablated (▲, dashed line) kidneys; means ± SD, *n* = 9, *P* > 0.05.

During an established hypoglycemia there were similar concentrations of PAH in the urine from nonablated and ablated kidneys in the course of five consecutive measurements performed every 15 min (Fig.3A). During the experiments the plasma concentrations of PAH were kept at about 15 *μ*g/mL (Fig.3B). Consequently, the side-dependent renal plasma flow (SRP) did not differ significantly after the unilateral ablation of the renal nerves (Fig.3C). Concurrently, the urine concentrations of inulin (Fig.4A) and glomerular filtration rates (GFR) (Fig.4B) did not differ significantly between nonablated and ablated kidneys. Taken together, these data indicate that, in relation to the plasma flow and the glomerular filtration, both of the pigs’ kidneys, control and denervated, performed highly similar.

**Figure 5:**
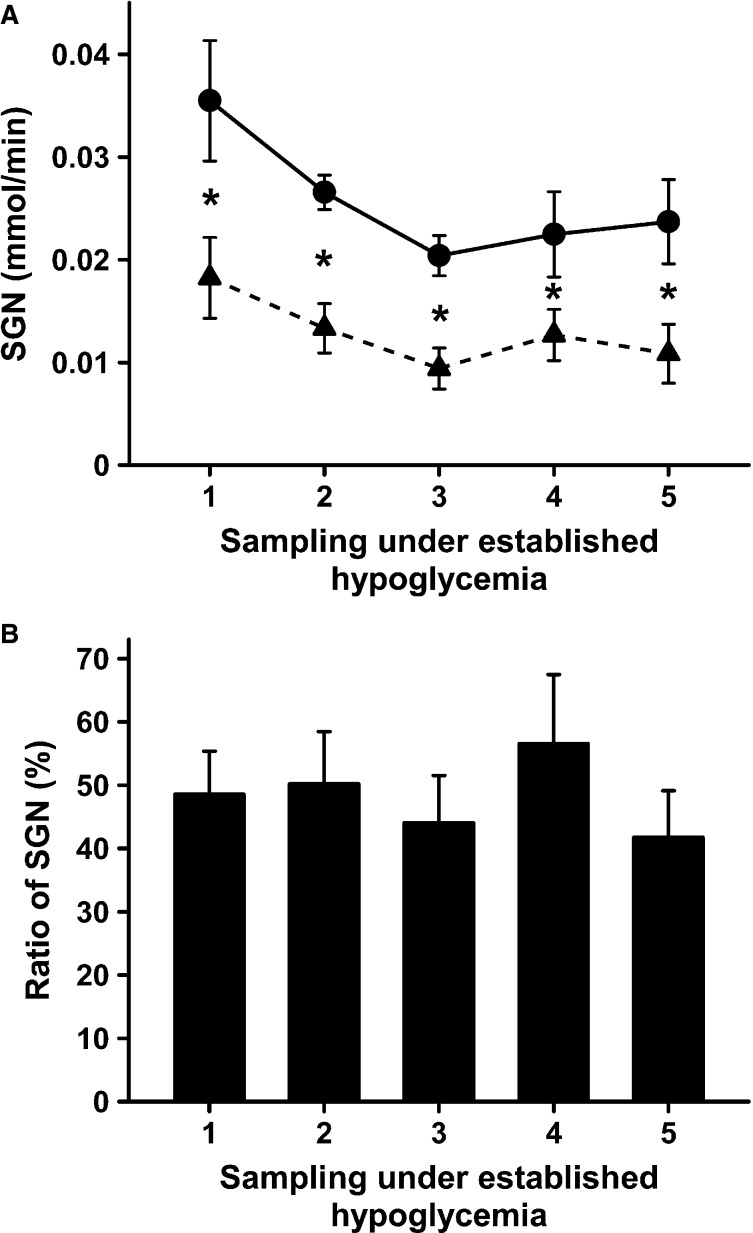
Side-dependent renal net glucose release (SGN) after unilateral ablation of renal nerves. Samples for SGN measurements were taken every 15 min during high-volume infusion induced hyperperfusion of the kidneys after hypoglycemia (blood glucose below 2 mmol/L) was established. (A) Significantly lower SGN (mmol/min) during severe hypoglycemia after unilateral ablation of renal nerves in nonablated (●, line) and ablated (▲, dashed line) kidneys; means ± SD, *n* = 9, *P* < 0.001 (*). SGN decreased significantly over time in both kidneys (linear mixed model; *P* = 0.036). (B) Ratios of SGNs of the ablated kidneys versus the nonablated kidneys [each in mmol/min] are given as percent values of the corresponding nonablated kidneys; means ± SD, *n* = 9.

**Figure 6:**
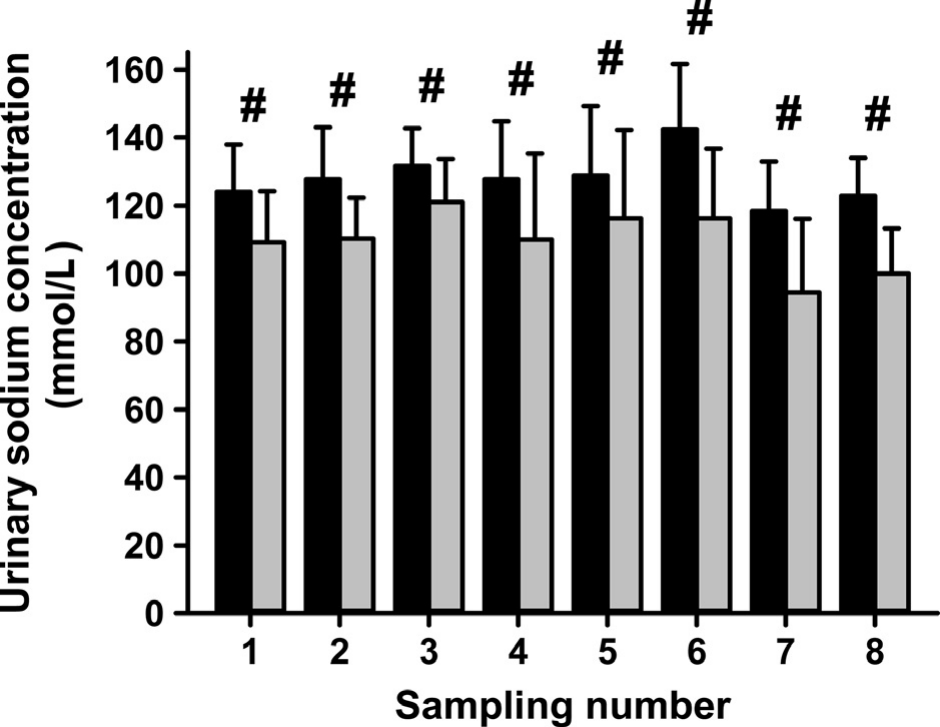
Urinary sodium concentrations after unilateral ablation of renal nerves. Samples for sodium excretion measurements were taken every 15 min during high-volume infusion induced hyperperfusion of the kidneys. During severe hypoglycemia significantly higher urinary sodium concentrations were measured in ablated (black bars) than in nonablated (gray bars) kidneys after unilateral ablation of renal nerves (linear mixed model; ^#^*P* = 0.019).

In the same experimental setting renal denervation had a significant negative effect (*P* < 0.001 for every sampling) on the side-dependent renal glucose release (SGN) during the established hypoglycemia (Fig.5A). A linear mixed effects model was adapted to compare the data of ablated and nonablated kidneys after hypoglycemia, confirming the reduction of gluconeogenesis caused by renal denervation (*P* = 0.036). The SGN of the ablated kidney decreased by 0.02 mmol/min and is proportional 43.3 ± 23.2% compared to the control (nonablated) kidney (Fig.5B). In this model, there was no significant (*P* = 0.354) interaction regarding measurement number or kidney side (ablated/nonablated), that is, the difference in SGN is nearly constant in all measurements (Fig.5) (*P* < 0.001) and decreased significantly over time in both kidneys (linear mixed model; *P* = 0.036). We also performed a detailed regression analysis at particular time points. In the first 15 min, SGN is significantly decreased by 0.01 mmol/min on average (*P* = 0.003). During the following 60 min the SGN stabilized in both groups (*P* = 0.871). In the experimental setting renal denervation had a significantly positive effect on the side-dependent urinary sodium concentration during the consecutive measurements, performed every 15 min (Fig.6). A linear mixed effects model was adapted to compare the data of ablated and nonablated kidneys after hypoglycemia, confirming the increase of urinary sodium concentration (*P* = 0.019).

## Discussion

An ablation of the renal autonomous nerves in pigs decreased SGN significantly during a severe hypoglycemia that was established and maintained during the course of the experiments by means of a hypoglycemic clamp. The SGN of the ablated kidney is nearly 55% lesser compared to the control (nonablated) kidney.

The SGN decreased over the whole time period of 75 min, but most significantly in the first time interval after 15 min of hypoglycemia had been maintained. This might be due to a depletion of substrates for the gluconeogenesis in the kidneys. Changes in intra- and intercellular metabolism, as well as utilization of substrates and kidney perfusion are potential reasons for this finding. However, the experimental design of this study was not set up to answer these questions.

We use the PAH clearance to estimate the SRP. The arterial PAH concentration during the course of the experimental hypoglycemia was kept under 15 *μ*g/mL, which is well below the saturating concentration of about 100 *μ*g/mL for the PAH secretion system of the proximal tubules, which consequently allows the use of the standard method for the calculation of the effective plasma flow. The GFR and SRP were not significantly lower in the ablated kidneys. Therefore, hemodynamic changes did not contribute to the effects of the renal nerve ablation on the glucose release during hypoglycemia. Previous studies show no changes in the GFR 1 h postprocedure in the kidneys of healthy people and in patients with chronic kidney diseases (Cockcroft and Gault 1976; Levey et al. 2009; Hering et al. 2012b; Kiuchi et al. 2013; Ott et al. 2013). The renal blood flow was similar to that obtained in other studies in humans (Ott et al. 2013). The stable SRP showed no postprocedure changes in the side-dependent perfusion and function during a hyperinfusion, which was necessary to maintain a controlled, stable blood pressure. In a pilot study, hyperinfusion was found to avoid renal failure, a possible complication due to the combined effects of anesthesia and hypoglycemia, and to ensure the continuation of a sufficient urine production.

The pathophysiological correlate in humans is not the well-managed diabetic patient, or the patient with still elevated blood glucose levels. The experimental setting of an induced hypoglycemia mimics an acute complication in patients with diabetes mellitus (both type 1 and type 2), which results from over dosage of insulin, some other antidiabetic drugs, or for example, stresses.

Therefore, we used a very deep hypoglycemia with blood glucose levels commonly found in affected patients. In order to avoid a too fast decrease of blood glucose levels throughout the course of the experiment, we induced hypoglycemia by repetitive administrations of insulin. The created acute situation reveals the involvement of the autonomous nervous system in the renal glucose mobilization during a severe hypoglycemia. A sufficient examination of the effect compared to the opposite side (respectively the other kidney) is only possible through a unilateral renal nerve ablation. The experimental setting ensures a repeatability of the experiment, even under these extreme conditions.

Initially, we apprehended that the mechanism would be hard to measure or that it would pose only 10–15% of the total glucose mobilization of the kidney. In pilot tests for the optimization of the experiment we established a hypoglycemia with a stable blood glucose concentration of 1.8 mmol/L in three animals. In so doing, it unfortunately became obvious that a steady administration of insulin and glucose 40% via perfusors leads to high variations in the renal glucose metabolization. It became apparent that even slight amounts of glucose administered during hypoglycemia lead to a cessation of the excretion of glucose from the kidneys, or even to glucose uptake.

The pigs used in the current study were healthy and were given no drugs affecting blood pressure, kidney function, or glucose metabolism, like catecholamines, prior to the experiments. In other studies, there was a decreased renal vascular resistance and an increased renal blood flow due to a reduced sympathetic tonus, which is known to be one of the major determinants of the GFR (Baylis and Brenner 1978; Kiuchi et al. 2013). We found no evidence of an increased renal blood flow to the ablated kidney. Therefore, our data are in agreement with the findings discussed farther above (Cockcroft and Gault 1976; Levey et al. 2009; Hering et al. 2012b; Kiuchi et al. 2013; Ott et al. 2013). Importantly, we found evidence for an increased sodium excretion by the nerve ablated kidney. This shows that the experimental setting of renal nerve ablation functions. Here again, our data are in agreement with the findings discussed farther above (DiBona 2005; Kopp 2015; Poss et al. 2015). Due to our chosen experimental setting no statement can be made on whether an altered glucose mobilization is caused by the renorenal reflex because we did not measure nerve activities (Kopp 2015).

The functional significance of the autonomic nervous system has recently attracted much attention in this field of research. Autonomic nerves are essential components of the endogenous system for maintaining energy homeostasis. In addition, this study and other investigations have recently demonstrated that the autonomic nervous system has a key role in transmitting metabolic information (Shimazu 1981; Woods 2004; Yamada et al. 2008). The hypothalamus in particular is a primary site of convergence and integration for redundant energy status signaling, which encompasses both central and peripheral neural inputs as well as hormonal and nutritional factors (Yamada and Katagiri 2007). These intertissue communication pathways are based on circulating humoral factors, including insulin and adipocytokines. These peptides also function outside the central nervous systems, influencing the activities of neurons, for example, the vagal afferent nerve which projects to the nucleus of the solitary tract in the brainstem (Shimazu 1981; Smith and Minson 2012; Schlaich et al. 2013).

Intra-abdominal tissues are innervated by both splanchnic (sympathetic) and vagal (parasympathetic) nerves. These nerve bundles consist of both efferent and afferent fibers. Detailed fiber count studies have revealed abdominal vagal and splanchnic nerves to comprise, respectively, of approximately 75 and 50% afferent fibers. Vagal afferents respond to specific chemical stimuli, the degree of physiological gut distention and nutrients, whereas splanchnic afferents carry information about noxious stimuli (Badman and Flier 2005). Numerous reports have described the important metabolic roles of the autonomic nervous systems in the gastrointestinal tract, pancreas, and liver (Shimazu 1981; Bartness and Bamshad 1998; Imai et al. 2006). The efferent parasympathetic innervation of intra-abdominal tissues is controversially discussed (Kreier et al. 2002).

The selection of the experimental setup with its undeniable advantages poses at the same time one of its limitations. A direct transfer of our findings on the therapeutic practice on humans is difficult – especially because long-term, chronical alterations are present in patients with diabetes mellitus and autonomous neuropathy, while our experiment is modeled on an acute situation (although a hypoglycemia poses an acute situation in this category of patients as well). Furthermore, we have used a unilateral nerve ablation in our experiment, while a bilateral renal nerve ablation is regularly practiced on humans. The advantage of our model is the reduced number of test animals and the reduction of the effects of interindividual differences between the animals.

In this study, we have demonstrated that the autonomic nervous system with the sympathetic fibers has a key role in the transmission of metabolic information to the kidneys. Autonomic nerves are essential components of the endogenous system for maintaining energy homeostasis during renal gluconeogenesis under insulin-induced hypoglycemia. The renal sympathetic nerve fibers around the renal arteries are a primary site of convergence and integration for redundant energy status signaling. The renal autonomic nervous system, as exemplified by these neuronal circuits, has an important role in regulating the energy metabolism.

## Conclusions

In summary, in pigs the ablation of renal autonomous nerves halves renal gluconeogenesis during severe hypoglycemia. Due to the effects of denervation on the glucose metabolism, the use of renal denervation must be considered as a possibly critical issue – at least for patients receiving antidiabetic medications. Therefore, patients should be made aware of this probable side effect.

## References

[b1] Agarwal R (2002). Rapid microplate method for PAH estimation. Am. J. Physiol. Renal. Physiol.

[b2] Badman MK, Flier JS (2005). The gut and energy balance: visceral allies in the obesity wars. Science.

[b3] Bartness TJ, Bamshad M (1998). Innervation of mammalian white adipose tissue: implications for the regulation of total body fat. Am. J. Physiol.

[b4] Baylis C, Brenner BM (1978). The physiologic determinants of glomerular ultrafiltration. Rev. Physiol. Biochem. Pharmacol.

[b5] Bhatt DL, Kandzari DE, O’Neill WW, D’Agostino R, Flack JM, Katzen BT (2014). A controlled trial of renal denervation for resistant hypertension. N. Engl. J. Med.

[b6] Care and Use of Laboratory Animals, Institute for Laboratory Animal Research (2011). Guide for the care and use of laboratory animals vol. 8.

[b7] Cersosimo E, Molina PE, Abumrad NN (1997). Renal glucose production during insulin-induced hypoglycaemia. Diabetes.

[b8] Cockcroft DW, Gault MH (1976). Prediction of creatinine clearance from serum creatinine. Nephron.

[b9] Constanzo L (2007). Physiology, vol. 4.

[b10] Davies JE, Manisty CH, Petraco R, Barron AJ, Unsworth B, Mayet J (2013). First-in-man safety evaluation of renal denervation for chronic systolic heart failure: primary outcome from REACH-Pilot study. Int. J. Cardiol.

[b11] Davis MI, Filion KB, Zhang D, Eisenberg MJ, Afilalo J, Schiffrin EL (2013). Effectiveness of renal denervation therapy for resistant hypertension: a systematic review and meta-analysis. J. Am. Coll. Cardiol.

[b12] DiBona GF (2005). Physiology in perspective: the Wisdom of the Body. Neural control of the kidney. Am. J. Physiol. Regul. Integr. Comp. Physiol.

[b13] Hering D, Esler MD, Schlaich MP (2012a). Effects of renal denervation on insulin resistance. Expert Rev. Cardiovasc. Ther.

[b14] Hering D, Mahfoud F, Walton AS, Krum H, Lambert GW, Lambert EA (2012b). Renal denervation in moderate to severe CKD. J. Am. Soc. Nephrol.

[b15] Imai J, Katagiri H, Yamada T, Ishigaki Y, Ogihara T, Uno K (2006). Cold exposure suppresses serum adiponectin levels through sympathetic nerve activation in mice. Obesity (Silver Spring).

[b16] Kiuchi MG, Maia GL, de Queiroz Carreira MA, Kiuchi T, Chen S, Andrea BR (2013). Effects of renal denervation with a standard irrigated cardiac ablation catheter on blood pressure and renal function in patients with chronic kidney disease and resistant hypertension. Eur. Heart J.

[b17] Kopp UC (2015). Role of renal sensory nerves in physiological and pathophysiological conditions. Am. J. Physiol. Regul. Integr. Comp. Physiol.

[b18] Kreier F, Fliers E, Voshol PJ, Van Eden CG, Havekes LM, Kalsbeek A (2002). Selective parasympathetic innervation of subcutaneous and intra-abdominal fat–functional implications. J. Clin. Invest.

[b19] Lambert GW, Straznicky NE, Lambert EA, Dixon JB, Schlaich MP (2010). Sympathetic nervous activation in obesity and the metabolic syndrome–causes, consequences and therapeutic implications. Pharmacol. Ther.

[b20] Levey AS, Stevens LA, Schmid CH, Zhang YL, Feldman AF, Castro HI (2009). A new equation to estimate glomerular filtration rate. Ann. Intern. Med.

[b21] Mahfoud F, Schlaich M, Kindermann I, Ukena C, Cremers B, Brandt MC (2011). Effect of renal sympathetic denervation on glucose metabolism in patients with resistant hypertension: a pilot study. Circulation.

[b22] Meyer C, Dostou JM, Gerich JE (1999). Role of the human kidney in glucose counterregulation. Diabetes.

[b23] Miller ER, Ullrey DE (1987). The pig as a model for human nutrition. Annu. Rev. Nutr.

[b24] Morales J, Schneider D (2014). Hypoglycaemia. Am. J. Med.

[b25] Noll G, Wenzel RR, Schneider M, Oesch V, Binggeli C, Shaw S (1996). Increased activation of sympathetic nervous system and endothelin by mental stress in normotensive offspring of hypertensive parents. Circulation.

[b26] Ott C, Janka R, Schmid A, Titze S, Ditting T, Sobotka PA (2013). Vascular and renal hemodynamic changes after renal denervation. Clin. J. Am. Soc. Nephrol.

[b27] Parati G, Esler M (2012). The human sympathetic nervous system: its relevance in hypertension and heart failure. Eur. Heart J.

[b28] Poss J, Ewen S, Schmieder RE, Muhler S, Vonend O, Ott C (2015). Effects of renal sympathetic denervation on urinary sodium excretion in patients with resistant hypertension. Clin. Res. Cardiol.

[b29] Reubi FC (1953). Glomerular filtration rate, renal blood flow and blood viscosity during and after diabetic coma. Circ. Res.

[b30] Roe JH, Epstein JH, Goldstein NP (1949). A photometric method for the determination of inulin in plasma and urine. J. Biol. Chem.

[b31] Schlaich MP, Schmieder RE, Bakris G, Blankestijn PJ, Bohm M, Campese VM (2013). International expert consensus statement: percutaneous transluminal renal denervation for the treatment of resistant hypertension. J. Am. Coll. Cardiol.

[b32] Schmieder RE, Redon J, Grassi G, Kjeldsen SE, Mancia G, Narkiewicz K (2012). ESH position paper: renal denervation - an interventional therapy of resistant hypertension. J. Hypertens.

[b33] Shimazu T (1981). Central nervous system regulation of liver and adipose tissue metabolism. Diabetologia.

[b34] Smith MM, Minson CT (2012). Obesity and adipokines: effects on sympathetic overactivity. J. Physiol.

[b35] Soveri I, Berg UB, Bjork J, Elinder CG, Grubb A, Mejare I (2014). Measuring GFR: a systematic review. Am. J. Kidney Dis.

[b36] Straznicky NE, Grima MT, Eikelis N, Nestel PJ, Dawood T, Schlaich MP (2011). The effects of weight loss versus weight loss maintenance on sympathetic nervous system activity and metabolic syndrome components. J. Clin. Endocrinol. Metab.

[b37] Sverrisdottir YB, Mogren T, Kataoka J, Janson PO, Stener-Victorin E (2008). Is polycystic ovary syndrome associated with high sympathetic nerve activity and size at birth?. Am. J. Physiol. Endocrinol. Metab.

[b38] Triplitt CL (2012). Understanding the kidneys’ role in blood glucose regulation. Am. J. Manag. Care.

[b39] Witkowski A, Prejbisz A, Florczak E, Kadziela J, Sliwinski P, Bielen P (2011). Effects of renal sympathetic denervation on blood pressure, sleep apnea course, and glycemic control in patients with resistant hypertension and sleep apnea. Hypertension.

[b40] Woods SC (2004). Gastrointestinal satiety signals I. An overview of gastrointestinal signals that influence food intake. Am. J. Physiol. Gastrointest. Liver Physiol.

[b41] Yamada T, Katagiri H (2007). Avenues of communication between the brain and tissues/organs involved in energy homeostasis. Endocr. J.

[b42] Yamada T, Oka Y, Katagiri H (2008). Inter-organ metabolic communication involved in energy homeostasis: potential therapeutic targets for obesity and metabolic syndrome. Pharmacol. Ther.

